# Evaluation of milk-based and egg-based semen extenders for extended storage of ram semen at 4°C

**DOI:** 10.1093/tas/txag018

**Published:** 2026-02-16

**Authors:** Emily R Schoelerman, Tyler J Weide, Karl C Kerns

**Affiliations:** Department of Animal Science, Iowa State University, Ames, IA, 50011, United States; Department of Animal Science, Iowa State University, Ames, IA, 50011, United States; Department of Animal Science, Iowa State University, Ames, IA, 50011, United States

**Keywords:** artificial insemination, liquid storage, ram semen, semen extender, zinc signature

## Abstract

Ram semen cryopreservation and liquid storage are essential tools for reproductive management in sheep, but remain limited by the sensitivity of sperm to cold shock and oxidative damage. Commercial extenders aim to preserve sperm function, yet differences in biochemical composition may influence their efficacy across key indicators of fertility. This study evaluated the effects of two commercial extenders, INRA96 and OviPlus, on ram sperm quality during liquid storage at 4°C over a 96-h period. Semen samples were collected from mature rams and diluted into each extender, then assessed at 0, 12, 24, 36, 48, 60, and 96 h post-collection for membrane integrity, acrosomal remodeling, reactive oxygen species (ROS), and zinc signatures using image-based flow cytometry (IBFC), as well as motility parameters measured with Computer Assisted Semen Analysis (CASA). While both extenders supported initial sperm viability, OviPlus had significantly less plasma membrane destabilization (timepoints 0, 12, 36, 48; *P* ≤ 0.03) and acrosomal damage (timepoints 12, 24, 36, 48, 60 and 96; *P* ≤ 0.002) across timepoints when compared to INRA96, as indicated by lower PI and PNA labeling. ROS levels increased over time in both groups but remained significantly lower in OviPlus (timepoints 0 and 36; *P* ≤ 0.01) preserved samples, suggesting a more protective oxidative environment. Zinc signature profiling revealed time-dependent shifts in capacitation status, with INRA96-treated sperm undergoing accelerated zinc efflux and increased transition into capacitated (Signature 3) states by 60 h, whereas OviPlus maintained a higher proportion of viable, uncapacitated sperm over the same interval. These findings demonstrate that extender formulation significantly influences ram sperm stability during liquid storage. OviPlus offered increased preservation of structural and functional sperm parameters, supporting its potential utility for extending the fertility window in ram artificial insemination protocols. Future research is needed to explore fertilization competency of stored ram sperm and extender-specific differences.

## Teaser

This study evaluates the effects of a milk-based (INRA96^®^96) and an egg-based (OviPlus) extender on liquid-stored ram sperm over 96 h.

## Introduction

Artificial insemination (AI) serves as an important strategy for accelerating genetic improvement of sheep herds around the world (Candappa and Bartlewski 2011; Alvarez et al. 2019). It enables the widespread distribution of superior sire genetics while mitigating biosecurity risks associated with natural mating. The adoption of AI in livestock production has facilitated enhanced reproductive efficiency, optimized breeding programs, and improved genetic selection strategies. Despite its advantages, AI has not been as widely adopted in the sheep industry, in part due to relatively poor fertility obtained when frozen semen is used for intracervical insemination.

Semen quality is critical to AI success, and preservation methods play a vital role in maintaining sperm viability and fertilizing potential. Cryopreservation remains the standard for long-term storage, offering indefinite preservation. However, the freeze-thaw process induces structural and functional damage, compromising sperm membrane and acrosome integrity, increasing reactive oxygen species (ROS), and decreasing motility, all detrimental to fertilizing potential. As a result, conception rates with frozen-thawed semen are often poor unless deposited intrauterine by laparoscopic procedure (Alvarez et al. 2019). Laparoscopic AI however, requires highly trained technicians which incur greater costs.

While post-thaw semen quality is often inferior to that of fresh semen, alternative storage methods, such as liquid-cooled storage, may mitigate some of these cryopreservation-induced detriments. Liquid storage of semen at refrigerated temperatures has been explored as a viable alternative to cryopreservation, particularly for short-term AI applications (Maxwell and Salamon 1993; Paulenz et al. 2002). Compared to frozen-thawed semen, liquid stored semen exhibits superior motility and membrane integrity (Maxwell and Salamon 1993). However, sperm viability declines over time, necessitating the use of protective extenders to mitigate oxidative damage, stabilize membranes, and preserve acrosomal function during storage.

Semen extenders are crucial in preserving sperm function by supplying antioxidants, energy substrates, and protection against loss of sperm membrane lipids (Maxwell and Salamon 1993; Salamon and Maxwell 2000; Foote et al. 2002; Bustani and Baiee 2021; Marini et al. 2023). Among extenders commonly used for ram semen storage, milk-based and egg yolk-based formulations have demonstrated efficacy in protecting sperm function during storage (Salamon and Maxwell 2000; Purdy 2006; Manjunath 2018). Milk-based extenders such as INRA96 contain casein micelles that stabilize sperm membranes through protein-protein interactions with seminal plasma proteins (Lusignan et al. 2011). In contrast, egg yolk-based extenders like OviPlus provide low-density lipoproteins (LDLs) that bind detrimental seminal proteins through lipid-protein interactions, as well as shield sperm from lipid peroxidation and (Manjunath et al. 2002; Bergeron et al. 2004).

Across livestock species, milk- and egg-yolk-based extenders have been evaluated under distinct semen preservation paradigms, and the depth of mechanistic understanding differs substantially by species and storage context. In cattle, the majority of foundational work comparing milk- and egg-derived protectants has focused on cryopreservation, where frozen semen is the dominant artificial insemination modality (Manjunath et al. 2002; Bergeron et al. 2004; Purdy 2006; Manjunath et al. 2009). These studies established that egg-yolk low-density lipoproteins (LDLs) and milk-derived casein micelles mitigate binder of sperm protein (BSP)-mediated lipid efflux, preserve membrane cholesterol, and stabilize sperm function during freezing and thawing (Bergeron et al. 2004; Lusignan et al. 2011; Manjunath 2018).

A smaller, but well-established, body of literature has also examined milk- and egg-based extenders for liquid storage of fresh bull semen, reflecting production systems in which short-term cooled semen is utilized (Purdy 2006; Manjunath 2018). In parallel, extensive applied research in swine and equine systems, which rely heavily on fresh and cooled semen, has evaluated diverse protein and lipid sources for extending semen lifespan, primarily emphasizing conventional fertility-associated endpoints such as motility, membrane integrity, and acrosome status (Salamon and Maxwell 2000; Purdy 2006; Bergeron et al. 2007).

In contrast, ovine artificial insemination often relies on refrigerated liquid semen, as frozen-thawed ram semen yields poor fertility following cervical insemination (Salamon and Maxwell 2000). Despite this reliance, ram-specific studies comparing milk- and egg-yolk-based extenders during liquid storage remain comparatively limited and have largely focused on bulk or endpoint measurements, including subjective or CASA-based motility, membrane integrity, acrosome status, and indirect indicators of oxidative damage such as lipid peroxidation (Paulenz et al. 2002; Hollinshead 2004 ; Khalifa and Lymberopoulos 2013 ; Stefanov et al. 2015; Arando 2019 ). Mechanistic assessments resolving oxidative stress pathways, zinc ion redistribution, and functionally distinct sperm subpopulations have not been incorporated into prior ram extender comparison studies.

The objective of this study was to evaluate the effects of commercially available INRA96 and OviPlus extenders on sperm viability and functional parameters during extended liquid storage at 4°C. Specifically, we assessed motility characteristics, plasma membrane and acrosomal integrity, ROS levels, and zinc signatures reflective of possible capacitation status during 96 h of liquid storage using Computer-Assisted Sperm Analysis (CASA) and Image-Based Flow Cytometry (IBFC). Understanding the differential effects of these extenders on sperm quality is essential for optimizing AI protocols in sheep breeding programs and improving reproductive outcomes.

## Material and methods

### Animal care and use statement

Ram semen samples used in this study were obtained through a collaborative partner of Iowa State University during routine semen collections conducted as part of standard production practices. These samples were not collected specifically for research purposes. Semen used was a byproduct of normal semen evaluation or production activities and no additional procedures were performed on the animals for research. Therefore, this study was exempt from review by the Iowa State University Institutional Animal Care and Use Committee (IACUC), in accordance with institutional policy. All procedures adhered to the *Guide for the Care and Use of Agricultural Animals in Research and Teaching* (Fass 2010) and followed standard industry protocols that ensure humane handling and welfare of the animals.

### Reagents

FluoZin-3, AM (FZ3; zinc probe) from ThermoFisher (F24195) was reconstituted with DMSO to a stock solution of 500 μM. Lectin PNA (A. hypogea/peanut agglutinin) conjugated to Alexa Fluor 594 (PNA-AF594) was from Invitrogen (L32459). Hoechst 33342 (H33342) from Calbiochem (382065) was reconstituted with ddH_2_O to a stock solution of 18 mM. Propidium iodide (PI) from Acros Organics (AC440300010) was reconstituted with ddH_2_O to a stock solution of 1 mg mL^−1^. CellROX Orange Reagent from Invitrogen (C10443) was reconstituted with DMSO to a stock solution of 2.5 μM.

### Extender preparation

INRA96 Equine Semen Extender made by IMV Technologies (Brooklyn Park, Minnesota, United States) was purchased and ready to use. INRA96 is composed of caseins, buffers, salts, sugars, ultrapure water, and antibiotics including sodium penicillin, gentamicin sulphate, and amphotericin B. OviPlus, fresh semen extender for small ruminants was made by and purchased from MiniTube (Minitüb GmbH, Tiefenbach, Germany). OviPlus concentrate is formulated with TRIS, citric acid, sugar, buffer, and antibiotics. Concentrate requires the addition of bi-distilled sterile water and fresh, locally sourced egg yolk. To obtain egg yolk, an egg was cracked, egg yolk and white were separated, egg yolk membrane was dried with a clean paper towel, and a syringe was inserted into the yolk to minimize contamination. OviPlus extender was mixed an hour prior to ram collection. Both extenders were slowly warmed to 37°C in a warming block before rams were collected.

### Modeling

Semen was collected from 4 to 5 mature crossbred rams for 2 wk and analyzed immediately upon arrival at the laboratory (0 h), 12, 24, 36, 48, 60 and 96 h following their arrival to the laboratory.

### Semen collection and processing

Semen was collected using an artificial vagina while rams mounted estrous ewes. Ejaculates with a volume of 1.2 mL or greater were immediately halved. One half of semen was extended with INRA96, the other half extended with OviPlus, both to a final concentration of 150 × 10^6^/mL. Extended semen samples were then placed in a Hamilton Biovet Equitaine to begin a slow controlled chilling process.

Samples were immediately transported to the Iowa State Department of Animal Sciences’ andrology laboratory for analysis. Timepoint 0 represents the time that samples arrived at the laboratory, approximately an hour after samples were collected. Upon arrival at the laboratory, semen was kept in a Hamilton Biovet Equitaine for 24 h for controlled chilling to approximately 4°C before being placed in 4°C refrigerator.

### Computer assisted semen analysis

100 μL of each sample was aliquoted into a 1.5 mL microcentrifuge tube and was further diluted with 200 μL of its respective extender. Diluted samples were mixed thoroughly with gentle pipetting then warmed at 37°C for 20 min. Consistent suspension of sperm in the warmed sample was ensured by gently pipetting the sample 10 times before 3 µL of each aliquot was loaded into a 20 µm chamber of a disposable 4-chamber slide from Minitube. Concentration and motility were then assessed using a Zeiss Axioscope 5 microscope (Carl Zeiss Microscopy, LLC, Oberkochen, Germany) fitted with a Basler ace ac2440-75uc camera (Basler AG, Ahrensburg, Germany) and a 10×/0.25 A-Plan Ph1-objective lens and using Minitube AndroVision software (Catalog Reference Module: 12,500/1000, Tiefenbach, Germany). The condenser with aperture diaphragm was set at 1 and the 6-position filter wheel was set at 2.

### Multiplex fluorescence labeling

A sample size of 5 million spermatozoa was added to non-capacitating media (modified TL-HEPES; [Kerns et al. 2018]) to be washed. Sperm were then incubated for 30 min with 1:2000 PNA-AF594, 1:1000 H33342, 1:1000 PI, 1:1000 CellROX, and 1:500 FZ3 at 37°C in non-capacitating media. The spermatozoa were then washed of probes once and resuspended in PBS without NaN_3_ to allow complete de-esterification of intracellular AM esters, as suggested by ThermoFisher’s FZ3 protocol.

### Image-based flow cytometry data acquisition

IBFC was performed using the ImageStream*®X* Mark II (AMNIS Cytek Biosciences, Fremont, CA, USA) image-based flow cytometer, fitted with a dual camera, 12 channel system, and a 40x objective lens magnification. The sheath fluid was PBS (without Ca^2+^ or Mg^2+^). The flow-core diameter and speed were 6 µm and 66 mm per second, respectively. The raw image data were acquired using INSPIRE software (AMNIS Cytek Biosciences). To produce the highest resolution, the camera setting was at 0.5 µm per pixel of the charge-coupled device. In INSPIRE IBFC data acquisition software, two brightfield channels were collected (channels 1 and 9), one FZ3 image (channel 2), one CellROX image (channel 3), one PI image (channel 5), one side scatter (SSC; channel 6), one H33342 (channel 7), and one PNA-AF594 image (channel 10) with a minimum of 10,000 spermatozoa collected. The following lasers were used: 405 nm laser for H33342 excitation at 10 mW, 488 nm laser for FZ3 excitation at 60 mW, 561 nm for CellROX excitation at 100 mW, 592 nm for PNA-AF594 excitation at 150 mW, and a 785 nm laser for side scatter (SSC) at 5 mW.

### IBFC data analysis

**Figure 1:**
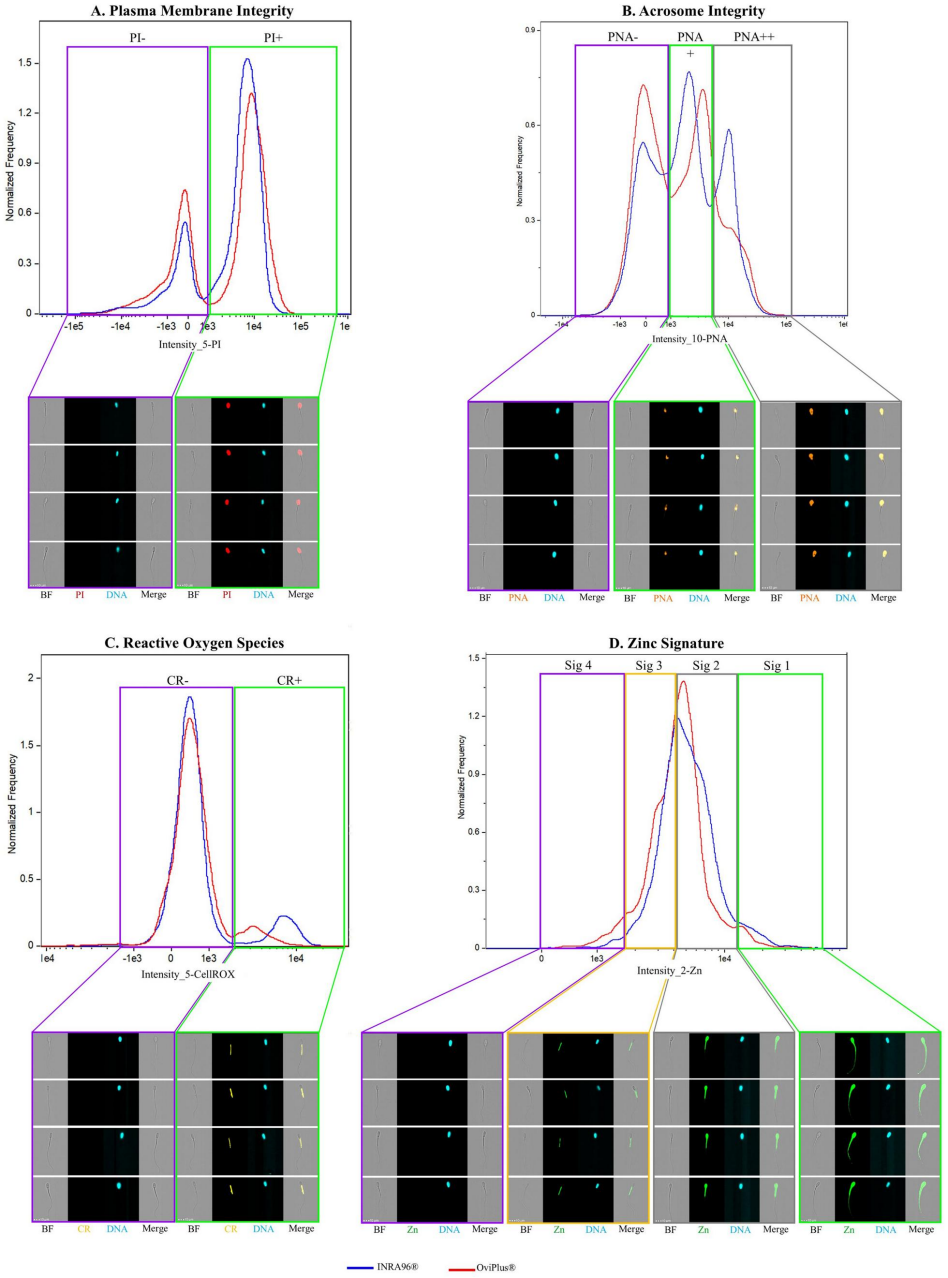
Representative examples of fluorescent gating strategies and corresponding image-based flow cytometry (IBFC) images used to assess ram sperm quality parameters. Each sperm cell was simultaneously imaged for brightfield (BF), plasma membrane integrity (propidium iodide; PI), acrosomal integrity (peanut agglutinin conjugated to Alexa Fluor 594; PNA), reactive oxygen species (CellROX Orange Reagent; CR), zinc distribution (FluoZin-3 am; Zn), DNA/nucleus (Hoechst 33342; DNA), and side scatter. IBFC image galleries display BF, the probe of interest, DNA, and a merged overlay (Merge) of BF and the probe. A) Plasma membrane integrity was evaluated using probe propidium iodide (PI), identifying two distinct populations: PI- sperm with intact membranes and PI+ sperm with damaged membranes. B) Acrosomal integrity was assessed using probe peanut agglutinin conjugated to Alexa Fluor 594 (PNA), revealing three populations: (1) PNA- sperm with intact acrosomes, (2) PNA+ sperm undergoing acrosomal remodeling, and (3) PNA++ sperm with extensive acrosomal modification. C) Reactive oxygen species (ROS) were detected using probe CellROX Orange Reagent (CR), which fluoresces upon oxidation, distinguishing CR− (low ROS) and CR+ (high ROS) sperm. D) Sperm zinc signatures were characterized using probe FluoZin-3 am, revealing four distinct Zn^2+^ localization patterns (Signatures 1–4) as reported by fluorescent activity: (1) high Zn^2+^ levels across the entire cell (non-capacitated sperm), (2) moderate Zn^2+^ levels in the head and midpiece (early capacitating sperm), (3) low Zn^2+^ levels restricted to the midpiece (mid-late capacitating sperm), and (4) absence of Zn^2+^ (dead or late-stage capacitated sperm). Blue lines represent semen extended in INRA96, and red lines represent ram semen extended in OviPlus.

The data were analyzed using IDEAS analysis software (AMNIS Cytek Biosciences), version 6.4. The gating approach used standard focus and single-cell gating calculations created by IDEAS software as previously described (Kerns et al. 2018). Representative examples of fluorescent gating strategies and the corresponding image-based flow cytometry (IBFC) imagery are shown in Fig. 1, demonstrating the gating fidelity and image clarity for plasma membrane integrity, acrosomal remodeling, reactive oxygen species production, and zinc signature localization.

### Statistical analysis

Statistical analyses were conducted in R (v4.2.2) using the nlme (v.3.1–168) (Pinheiro et al. 2023) and emmeans (v.1.11.0) (Lenth et al. 2025) packages to evaluate the effects of treatment and timepoint on the measured parameters. A linear mixed-effects model was fitted. In this model, Treatment, Time, and their interaction were included as fixed effects to estimate population-level differences across conditions, while Boar was included as a random effect to account for repeated measures and variability attributable to individual animals. Heterogeneous variances across treatment groups were accommodated using a variance structure in the model. Model assumptions were assessed for each fitted model. All data points were retained for analysis to reflect the full biological variability of the dataset; no outliers were excluded. Post hoc pairwise comparisons between groups were performed using the emmeans package to compute estimated marginal means (least-squares means). Tukey’s Honestly Significant Difference (HSD) method was applied to adjust for multiple comparisons. Group differences were visualized using bar charts using the ggplot2 (v.3.5.1) (Wickham et al. 2016) package with error bars representing the standard error of the mean (SEM). Statistical significance was assessed at *P* < 0.05, and results are reported with corresponding estimated marginal means and 95% confidence intervals.

## Results

### Plasma membrane integrity

**Figure 2:**
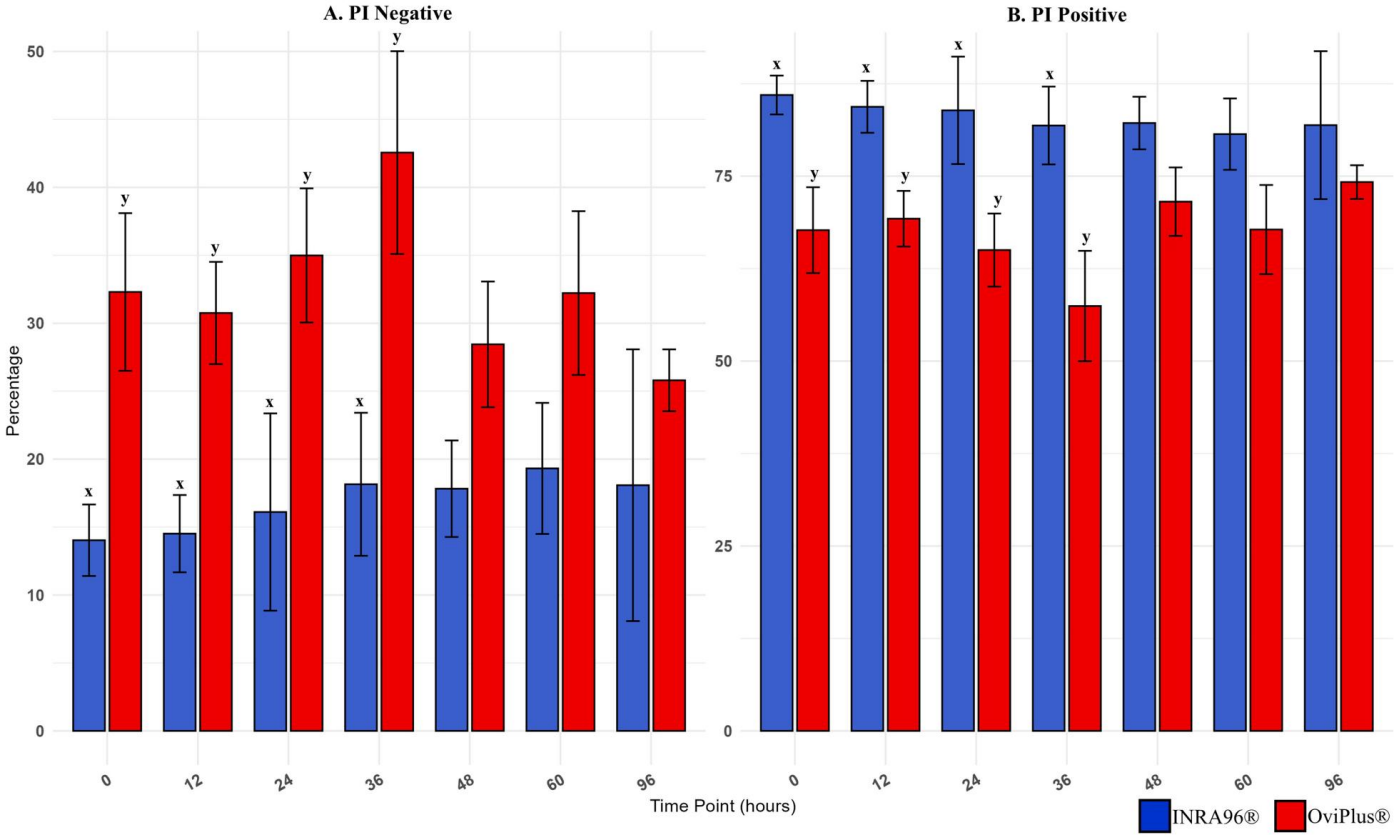
Effect of treatment on PI across all timepoints. Membrane changes were detected by IBFC using fluorescent PI. Two distinct populations were identified: A) PI-negative sperm cells with intact plasma membranes (PI−) and B) PI-positive sperm cells with compromised plasma membranes (PI+). Values with different superscripts (x, y) indicate significant differences between extenders at a given timepoint (*P* < 0.05). Blue bars represent semen extended in INRA96, and red bars represent semen extended in OviPlus. Bar height represents the mean; error bars represent standard deviation.

Plasma membrane integrity was assessed using propidium iodide (PI) which is taken up exclusively by cells with a compromised/remodeled plasma membrane (Garner et al. 1994; Davies and Hughes 2000; Kerns et al. 2018), distinguishing live (PI-negative) from membrane-compromised (PI-positive) sperm. The percentage of PI-positive cells differed significantly between treatments at 0, 12, 24, and 36 h post collection (*P* ≤ 0.3) and tended to be significant at 48 and 60 h (*P* ≤ 0.9; Table 1 and Fig. 2). INRA96 treated samples exhibited a greater proportion of membrane compromised cells compared to OviPlus, with no significant differences between time points within each treatment.

**Table 1:** Plasma membrane integrity.

	INRA96		OviPlus	
	PI+	PI−	PI+	PI−
**0 HR**	85.97 ± 7.89^x^	14.03 ± 17.40^x^	67.70 ± 7.89^y^	32.30 ± 17.40^y^
**12 HR**	84.37 ± 10.53^x^	14.51 ± 11.29^x^	69.25 ± 8.53^y^	30.75 ± 11.29^y^
**24 HR**	83.90 ± 21.78^x^	16.11 ± 14.81^x^	65.01 ± 21.78^y^	34.99 ± 14.81^y^
**36 HR**	81.84 ± 15.79^x^	18.15 ± 22.39^x^	57.44 ± 15.79^y^	42.56 ± 22.39^y^
**48 HR**	82.18 ± 10.66	17.82 ± 13.87	71.55 ± 10.66	28.45 ± 13.87
**60 HR**	80.68 ± 14.47	19.32 ± 18.08	67.78 ± 14.47	32.22 ± 18.08
**96 HR**	81.90 ± 29.99	18.08 ± 6.83	74.20 ± 29.99	25.80 ± 6.83

Membrane changes were detected by IBFC using fluorescent propidium iodide (PI). Two distinct populations were identified: PI-negative sperm cells with intact plasma membrane (PI−) and PI-positive sperm cells with compromised plasma membranes (PI+). No significant differences were observed between timepoints within INRA96 treated samples (*P* >0.05). No significant differences were observed between timepoints within OviPlus treated samples (*P* > 0.05). Values with different superscripts (x, y) indicate significant differences between extenders at a given timepoint (*P* < 0.05). Data are presented as mean ± standard deviation (SD).

### Acrosomal remodeling

**Figure 3:**
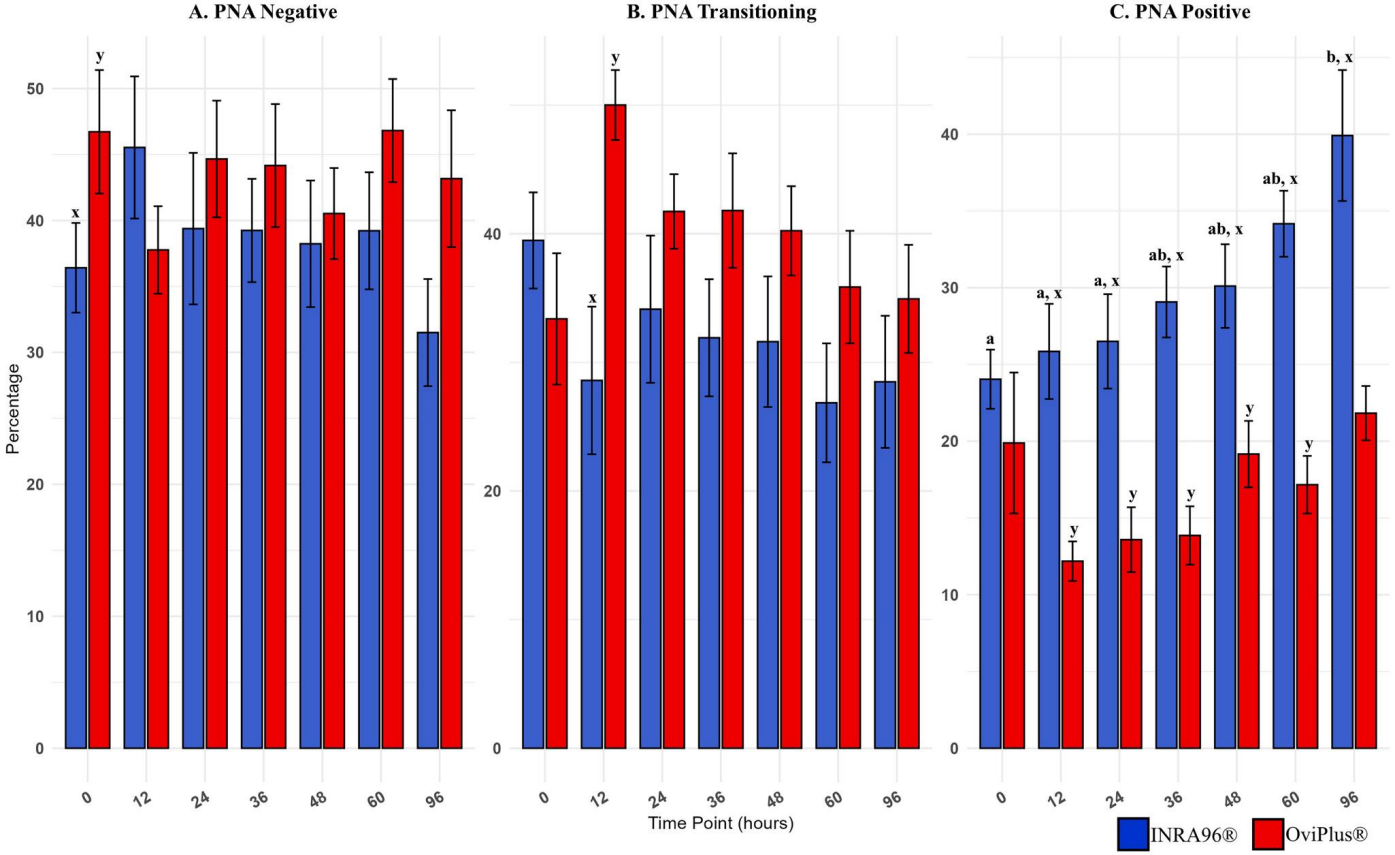
Effect of treatment on PNA across all timepoints. Acrosomal remodeling was evaluated using peanut agglutinin conjugated to alexa fluor 594 (PNA-594). Three distinct sperm populations were identified: A) PNA-negative sperm (PNA−) with intact acrosomes, B) PNA-transitioning (PNA+) sperm, and C) PNA-positive (PNA++) sperm with extensive acrosomal modification. Values with different lowercase subscripts (a, b, c) indicate significant differences between timepoints within INRA96 treated samples (*P* < 0.05). Values with different superscripts (x, y) indicate significant differences between extenders at a given timepoint (*P* < 0.05). Blue bars represent semen extended in INRA96, and red bars represent semen extended in OviPlus. Bar height represents the mean; error bars represent standard deviation.

Acrosomal remodeling was evaluated using peanut agglutinin conjugated to Alexa Fluor 594 (PNA-594). Three distinct sperm populations were identified: (i) PNA-negative sperm with intact acrosomes, (ii) PNA-transitioning sperm, and (iii) PNA-positive sperm with extensive acrosomal modification. The percentage of PNA-positive cells was significantly lower at 0, 12, and 24 h compared to 96 h (*P* ≤ 0.02) for semen extended in INRA96 (Table 2 and Fig. 3). Fewer cells tended to display modified acrosomes at 0 and 12 h compared to 60 h, and at 36 h compared to 96 h (*P* ≤ 0.09). No time-dependent differences were observed in OviPlus extended samples. Between treatments, INRA96 samples exhibited significantly greater percentages of PNA-positive sperm at time points 12, 24, 36, 48, 60, 96 h compared to OviPlus (*P* ≤ 0.002). Additionally, PNA-transitioning cells were significantly lower in INRA96 samples at 12 h (*P* = 0.001), while the proportion of PNA-negative cells was significantly lower at 0 h and tended to be higher at 96 h (*P* ≤ 0.05).

**Table 2:** Acrosome integrity.

	INRA96			OviPlus		
	PNA++	PNA+	PNA−	PNA++	PNA+	PNA−
**0 HR**	24.04 ± 10.19^a^	39.48 ± 14.04	36.41 ± 11.21^x^	19.88 ± 15.31	33.38 ± 5.78	46.72 ± 13.77^y^
**12 HR**	25.85 ± 16.15^ax^	28.59 ± 9.94^x^	45.53 ± 17.21	12.19 ± 8.15^y^	50.01 ± 9.30^y^	37.77 ± 3.86
**24 HR**	26.5 ± 17.22^ax^	34.13 ± 13.27	39.38 ± 17.17	13.59 ± 8.69^y^	41.73 ± 9.23	44.66 ± 6.33
**36 HR**	29.07 ± 11.74^abx^	31.91 ± 13.98	39.24 ± 13.66	13.86 ± 13.33^y^	41.8 ± 6.93	44.16 ± 5.69
**48 HR**	30.11 ± 14.39^abx^	31.61 ± 10.35	38.23 ± 15.23	19.16 ± 10.41^y^	40.23 ± 8.17	40.52 ± 6.48
**60 HR**	34.17 ± 13.31^abx^	26.85 ± 11.72	39.22 ± 13.87	17.17 ± 13.11^y^	35.85 ± 6.45	46.81 ± 5.63
**96 HR**	39.92 ± 12.18^bx^	28.49 ± 15.55	31.5 ± 15.40	21.83 ± 12.61^y^	34.93 ± 12.80	43.17 ± 5.31

Acrosomal remodeling was evaluated using peanut agglutinin conjugated to Alexa Fluor 594 (PNA-594). Three distinct sperm populations were identified: (1) PNA-negative sperm with intact acrosomes (PNA−), (2) PNA-transitioning sperm (PNA+), and (3) PNA-positive sperm with extensive acrosomal modification (PNA++). Values with different lowercase subscripts (a & b) indicate significant differences between timepoints within INRA96 treated samples (*P* < 0.05). No significant differences were observed between timepoints within OviPlus treated samples (*P* > 0.05). Values with different superscripts (x, y) indicate significant differences between extenders at a given timepoint (*P* < 0.05). Data are presented as mean ± standard deviation (SD).

### Reactive oxygen species (ROS) production

**Figure 4:**
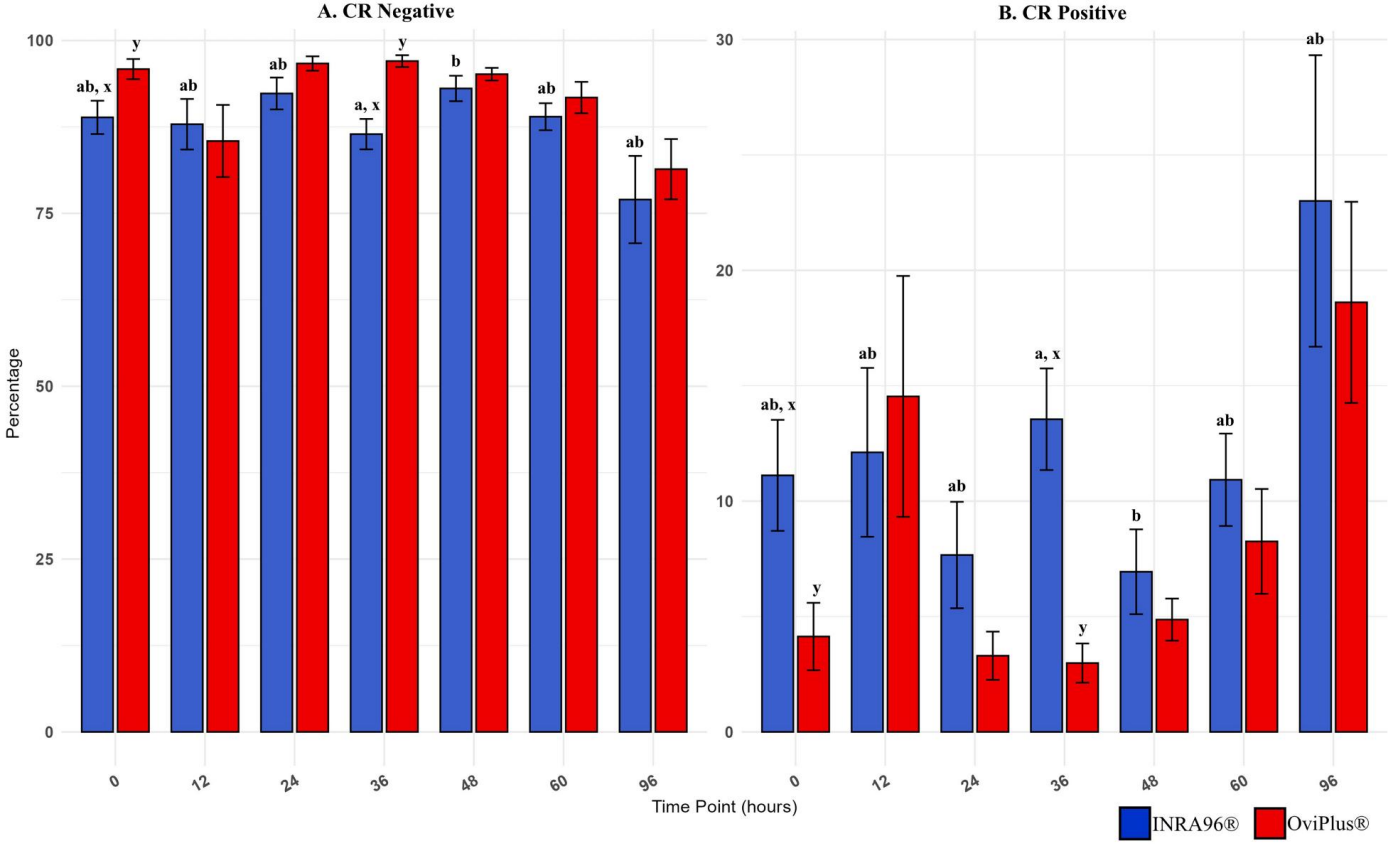
Effect of treatment on CellROX orange reagent across all timepoints. ROS were quantified using CellROX orange reagent, which fluoresces upon oxidation by reactive oxygen species. A) Sperm populations with low levels of ROS (CR−) and B) high levels of ROS (CR+) were identified. Values with different lowercase subscripts (a, b, c) indicate significant differences between timepoints within INRA96 treated samples (*P* < 0.05). Values with different superscripts (x, y) indicate significant differences between extenders at a given timepoint (*P* < 0.05). Blue bars represent semen extended in INRA96, and red bars represent semen extended in OviPlus. Bar height represents the mean; error bars represent standard deviation.

ROS levels were quantified using CellROX Orange Reagent (CR), which fluoresces upon oxidation by reactive oxygen species. CR-positive (high ROS) and CR-negative (low ROS) sperm populations were identified. ROS levels were significantly elevated in INRA96 samples at 36 h compared to 48 h (*P* = 0.04), with a non-significant increase at 48 h compared to 96 h (*P* = 0.08) (Table 3 and Fig. 4). No significant time-dependent differences were noted in OviPlus samples. Between treatments, OviPlus samples had significantly lower ROS levels at 0 and 36 h (*P* ≤ 0.01), with a trend toward lower ROS at 24 h (*P* = 0.09) compared to INRA96.

**Table 3:** Reactive oxygen species abundance.

	INRA96		OviPlus	
	CR+	CR−	CR+	CR−
**0 HR**	11.12 ± 7.22^abx^	88.88 ± 7.22^abx^	4.13 ± 4.38^y^	95.86 ± 4.38^y^
**12 HR**	12.11 ± 10.97^ab^	87.89 ± 10.97^ab^	14.54 ± 15.66	85.46 ± 15.66
**24 HR**	7.66 ± 6.92^ab^	92.33 ± 6.91^ab^	3.30 ± 3.13	96.67 ± 3.12
**36 HR**	13.55 ± 6.60^ax^	86.45 ± 6.60^ax^	2.98 ± 2.55^y^	97.02 ± 2.55^y^
**48 HR**	6.94 ± 5.51 b	93.06 ± 5.51^b^	4.87 ± 2.74	95.13 ± 2.73
**60 HR**	10.93 ± 6.01^ab^	88.97 ± 5.85^ab^	8.26 ± 6.81	91.74 ± 6.81
**96 HR**	23.01 ± 18.94^ab^	76.99 ± 18.94^ab^	18.61 ± 13.07	81.39 ± 13.07

ROS were quantified using CellROX Orange Reagent (CR), which fluoresces upon oxidation by reactive oxygen species. Sperm populations with high levels of ROS (CR+) and low levels of ROS (CR−) were identified. Values with different lowercase subscripts (a & b) indicate significant differences between timepoints within INRA96 treated samples (*P* < 0.05). No significant differences were observed between timepoints within OviPlus treated samples (*P* >0.05). Values with different superscripts (x, y) indicate significant differences between extenders at a given timepoint (*P* < 0.05). Data are presented as mean ± standard deviation (SD).

### Zinc signature patterns

Sperm zinc signatures, assessed using FluoZin-3 am, provided insights into potential capacitation status. Four distinct zinc (Zn^2+^) patterns (further referred to as zinc signatures 1–4) were identified as previously described by Kerns et al: (i) high levels of Zn^2 +  ^localized across the entire sperm, (ii) moderate Zn^2 +  ^in the head and midpiece (indicative of capacitating spermatozoa), (iii) Zn^2+^ localized to the midpiece (associated with capacitated spermatozoa) and (iv) absence of Zn^2+^, indicative of dead or dying capacitated sperm (Kerns et al. 2018).

**Figure 5:**
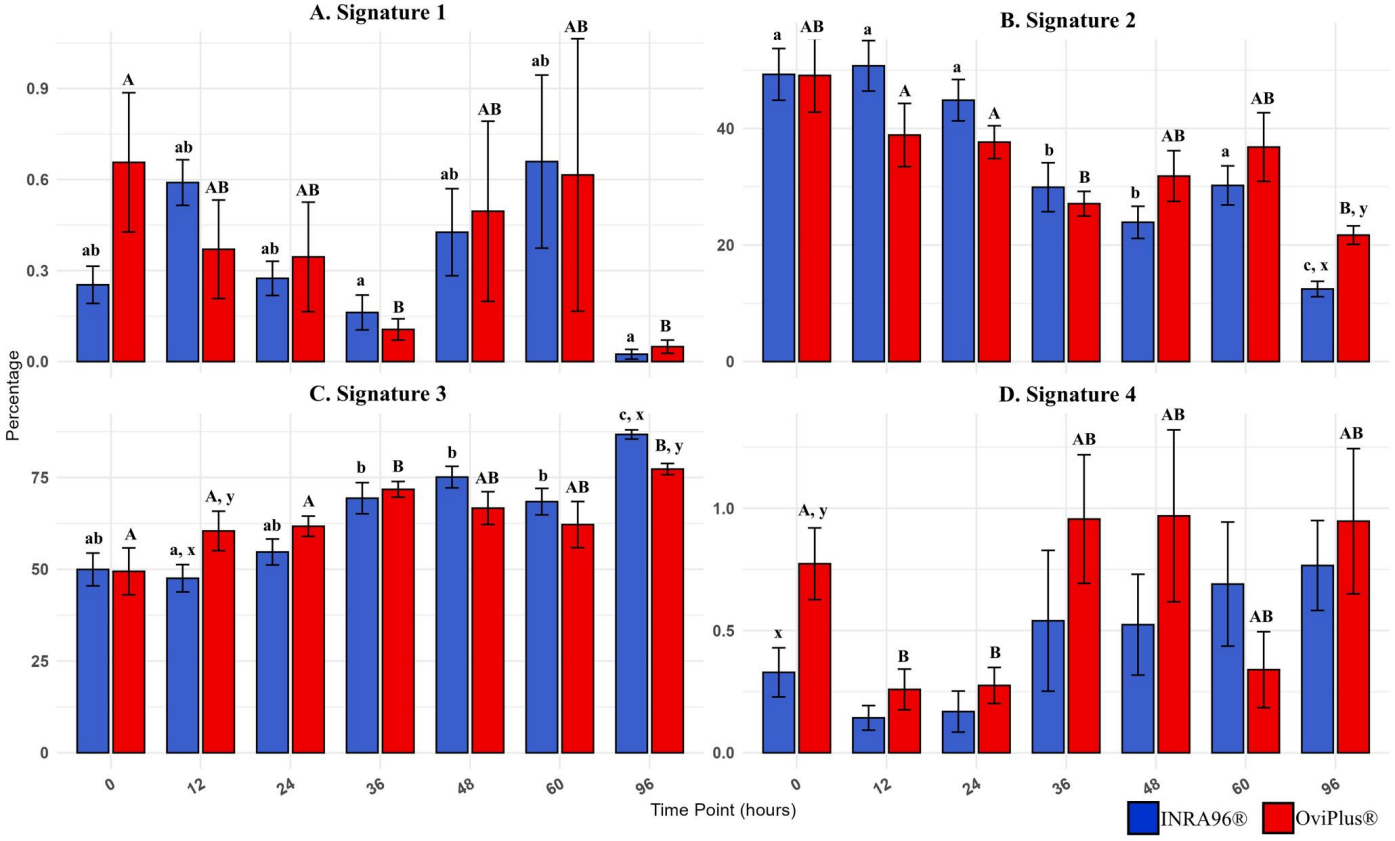
Effect of treatment on zinc signature across all timepoints. Sperm zinc signatures assessed using FluoZin-3 am, provided insights into potential capacitation status. Four distinct zinc (Zn^2+^) patterns (further referred to as zinc signatures 1–4) were identified: A) (signature 1) high levels of Zn^2+^ localized across the entire cell, B) (signature 2) moderate Zn^2+^ in the head and midpiece, C) (signature 3) Zn^2+^ localized to the midpiece and D) (signature 4) absence of Zn^2+^. Values with different lowercase subscripts (a, b, c) indicate significant differences between timepoints within INRA96 treated samples (*P* < 0.05). Values with different uppercase subscripts (A, B, C) indicate significant differences between timepoints within OviPlus treated samples (*P* < 0.05). Values with different superscripts (x, y) indicate significant differences between extenders at a given timepoint (*P* < 0.05). Blue bars represent semen extended in INRA96, and red bars represent semen extended in OviPlus. Bar height represents the mean; error bars represent standard deviation.

In INRA96 samples, Signature 1 sperm were significantly more abundant at 12 h than at 36 and 96 h (*P* ≤ 0.02; Table 4 and Fig. 5). Signature 2 sperm were significantly more abundant at 0, 12, and 24 h compared to 36, 48 and 96 (*P* ≤ 0.05). Additionally, there were significantly more Signature 2 sperm at 36, 48 and 60 compared to timepoint 96 (*P* ≤ 0.05). There tended to be more Signature 2 sperm a timepoint 12 compared to 60 (*P* = 0.05). Signature 3 sperm were significantly fewer at 0, 12 and 24 h compared to 36 (*P* ≤ 0.05), 48 (*P* ≤ 0.005), and 96 (*P* < 0.0001). Furthermore, there were fewer Signature 3 cells at timepoint 12 than 60 (*P* = 0.0497); as well as timepoints 36 and 60 compared to 96 h (*P* ≤ 0.02). There tended to be less Signature 3 cells at 48 h compared to 96 h (*P* = 0.05). Signature 4 cells tended to be less frequent at 12 and 24 h compared to 96 h (*P* ≤ 0.09).

**Table 4:** Zinc signatures.

	INRA96			
	Signature 1	Signature 2	Signature 3	Signature 4
**0 HR**	0.25 ± 0.18^ab^	49.28 ± 13.31^a^	49.93 ± 13.41^ab^	0.33 ± 0.30^x^
**12 HR**	0.59 ± 0.23^ab^	50.74 ± 12.98^a^	47.53 ± 11.17^ax^	0.14 ± 0.15
**24 HR**	0.27 ± 0.17^ab^	44.84 ± 10.65^a^	54.7 ± 10.61^ab^	0.17 ± 0.25
**36 HR**	0.16 ± 0.17^a^	29.91 ± 12.59^b^	69.34 ± 12.73^b^	0.54 ± 0.86
**48 HR**	0.43 ± 0.43^ab^	23.9 ± 8.27^b^	75.09 ± 8.79^b^	0.52 ± 0.62
**60 HR**	0.66 ± 0.86^ab^	30.23 ± 10.05^a^	68.41 ± 10.85^b^	0.69 ± 0.76
**96 HR**	0.02 ± 0.05^a^	12.46 ± 3.97^cx^	86.71 ± 3.73^cx^	0.77 ± 0.55
	**OviPlus**			
**0 HR**	0.66 ± 0.69^A^	49.09 ± 18.89^A^	49.42 ± 19.15^A^	0.77 ± 0.44^Ay^
**12 HR**	0.37 ± 0.49^AB^	38.87 ± 16.27^A^	60.43 ± 16.14^Ay^	0.26 ± 0.25^B^
**24 HR**	0.35 ± 0.54^AB^	37.65 ± 8.43^A^	61.71 ± 8.30^A^	0.28 ± 0.22^B^
**36 HR**	0.11 ± 0.11^B^	27.09 ± 6.34^B^	71.75 ± 6.43^B^	0.96 ± 0.79^AB^
**48 HR**	0.50 ± 0.89^AB^	31.83 ± 13.03^AB^	66.65 ± 13.40^AB^	0.97 ± 1.05^AB^
**60 HR**	0.62 ± 1.35^AB^	36.80 ± 17.67^AB^	62.16 ± 18.86^AB^	0.34 ± 0.47^AB^
**96 HR**	0.05 ± 0.07^B^	21.70 ± 4.75^By^	77.28 ± 4.59^By^	0.95 ± 0.89^AB^

Sperm zinc signatures assessed using FluoZin-3 am, provided insights into potential capacitation status. Four distinct zinc (Zn²⁺) patterns (further referred to as zinc signatures 1–4) were reported by fluorescent activity: (1) high Zn²⁺ levels localized across the entire sperm (non-capacitated sperm), (2) moderate Zn²⁺ levels in the head and midpiece (early stage capacitating sperm), (3) low Zn²⁺ levels restricted to the midpiece (mid-late capacitating sperm) and (4) absence of Zn²⁺ (dead or late-stage capacitated sperm). Values with different lowercase subscripts (a, b, c) indicate significant differences between timepoints within INRA96 treated samples (*P* < 0.05). Values with different uppercase subscripts (A & B) indicate significant differences between timepoints within OviPlus treated samples (*P* < 0.05). Values with different superscripts (x, y) indicate significant differences between extenders at a given timepoint (*P* < 0.05). Data are presented as mean ± standard deviation (SD).

In OviPlus samples, Signature 1 sperm was significantly more abundant at 0 h compared to 36 and 96 h (*P* = 0.01). The proportion of Signature 2 cells was significantly higher at 0 h compared to 36 and 96 h (*P* ≤ 0.01) and at 12 and 24 h compared to 96 h (*P* ≤ 0.02). A trend toward increased Signature 2 cells was observed at 60 h relative to 96 h (*P* = 0.05). Signature 3 cells were significantly increased at timepoint 36 compared to 0 (*P* = 0.01) and at hour 96 compared to hour 0, 12 and 24 (*P* < 0.0001). Signature 3 cells tended to be more abundant at timepoint 96 compared to 60 (*P* = 0.08). Signature 4 cell percentages were significantly lower at 12 and 24 h compared to 0 h (*P* ≤ 0.03).

Between treatments, INRA96 samples had significantly fewer Signature 3 cells at 12 h (*P* = 0.05), fewer Signature 2 cells and more Signature 3 cells at timepoint 96 (*P* < 0.0001), and at timepoint 0, INRA96 had fewer Signature 4 cells (*P* = 0.02). At timepoint 12 INRA96 tended to have more Signature 2 cells (*P* = 0.09), and at 0 h INRA96 tended to have increased Signature 1 sperm (*P* = 0.08) compared to OviPlus.

### Sperm motility parameters

Computer-assisted sperm analysis (CASA) was used to evaluate motility parameters.

### Total motility

**Figure 6:**
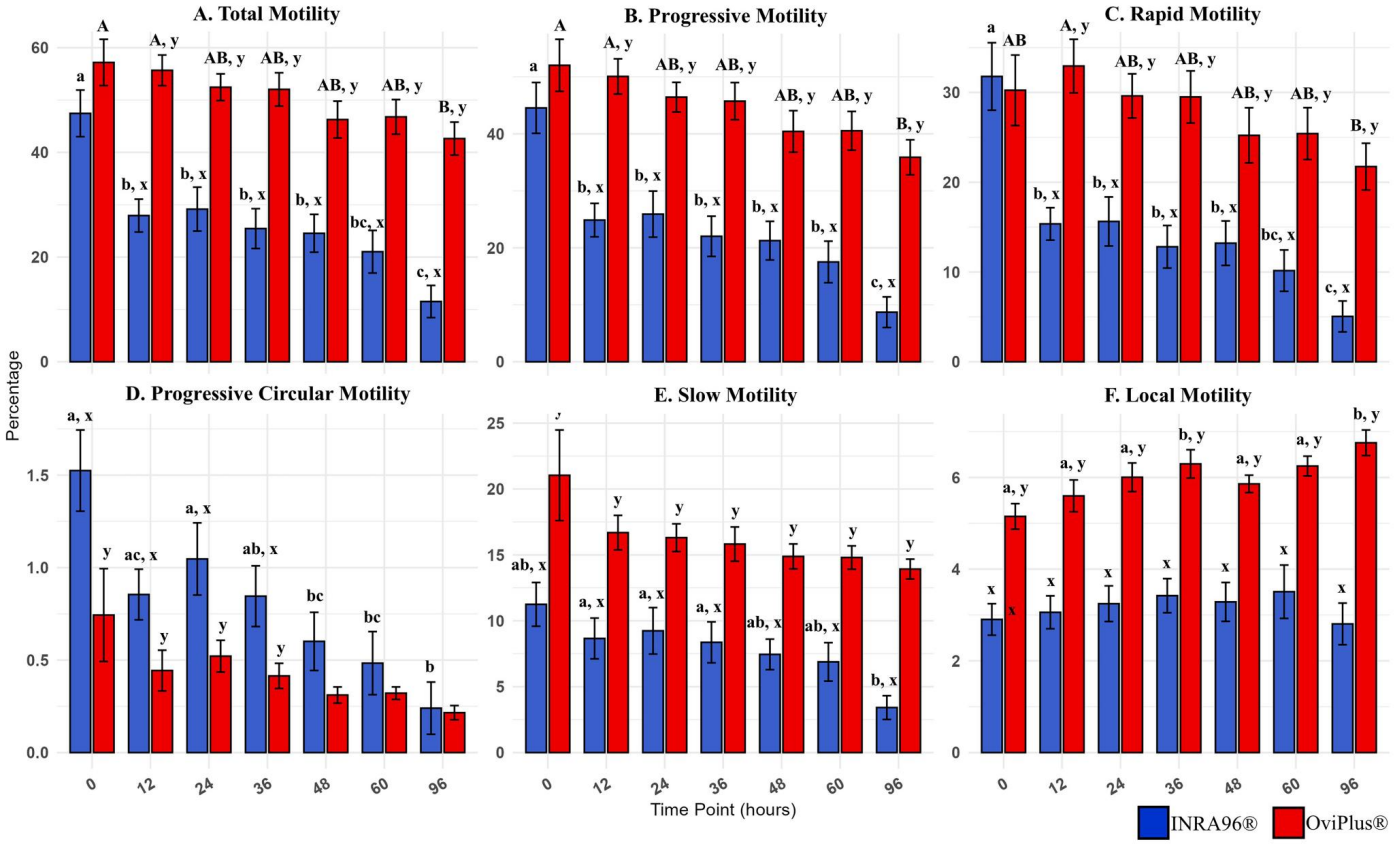
Effect of treatment on motility metrics across all timepoints. Computer assisted semen analysis (CASA) was used to assess A) Total motility, B) progressive motility, C) rapid motility, D) progressive circular motility, E) slow motility, F) local motility. Values with different lowercase subscripts (a, b, c) indicate significant differences between timepoints within INRA96 treated samples (*P* < 0.05). Values with different uppercase subscripts (A, B, C) indicate significant differences between timepoints within OviPlus treated samples (*P* < 0.05). Values with different superscripts (x, y) indicate significant differences between extenders at a given timepoint (*P* < 0.05). Blue bars represent semen extended in INRA96, and red bars represent semen extended in OviPlus. Bar height represents the mean; error bars represent standard deviation.

In INRA96 samples, total motility was significantly higher at 0 h than at all subsequent time points (*P* ≤ 0.02; Table 5 and Fig. 6A). Time points 12, 24, 36 and 48 h exhibited higher total motility than 96 h (*P* ≤ 0.03). OviPlus samples exhibited more stable total motility, with only hours 0 and 12 differing significantly from hour 96 (*P* = 0.05). Between treatments, total motility was similar at 0 h, but OviPlus exhibited higher total motility at all subsequent time points (*P* < 0.0001).

**Table 5:** Total motility.

	INRA96 (%)	OviPlus (%)
**0 HR**	47.46 ± 13.35^a^	57.18 ± 13.29^A^
**12 HR**	27.93 ± 9.43^bx^	55.67 ± 8.80^Ay^
**24 HR**	29.16 ± 12.59^bx^	52.45 ± 7.66^ABy^
**36 HR**	25.45 ± 11.40^bx^	52.04 ± 9.53^ABy^
**48 HR**	24.55 ± 10.84^bx^	46.28 ± 10.54^ABy^
**60 HR**	21.03 ± 12.20^bcx^	46.79 ± 9.91^ABy^
**96 HR**	11.51 ± 9.22^cx^	42.64 ± 9.45^By^

Values with different lowercase subscripts (a, b, c) indicate significant differences between timepoints within INRA96 treated samples (*P* < 0.05). Values with different uppercase subscripts (A & B) indicate significant differences between timepoints within OviPlus treated samples (*P* < 0.05). Values with different superscripts (x, y) indicate significant differences between extenders at a given timepoint (*P* < 0.05). Data are presented as mean ± standard deviation (SD).

### Progressive motility

Progressive motility followed a similar pattern as total motility, with INRA96 samples displaying significantly higher percentages of progressively motile sperm at timepoint 0 compared to all later time points (*P* ≤ 0.02; Table 6 and Fig. 6B). Furthermore, timepoints 12, 24, 36, and 48 exhibited significantly more progressively motile sperm compared to 96 (*P* ≤ 0.03). OviPlus samples exhibited significantly higher progressive motility at 0 and 12 h compared to 96 h (*P* ≤ 0.02). No differences were observed between treatments at 0 h, however OviPlus maintained significantly higher progressive motility compared to INRA96 12 h onward (*P* < 0.0001).

**Table 6:** Progressive motility.

	INRA96 (%)	OviPlus (%)
**0 HR**	44.55 ± 13.36^a^	52.03 ± 13.70^A^
**12 HR**	24.87 ± 8.81^bx^	50.08 ± 9.28^Ay^
**24 HR**	25.92 ± 12.14^bx^	46.44 ± 7.78^ABy^
**36 HR**	22.02 ± 10.57^bx^	45.74 ± 9.77^ABy^
**48 HR**	21.26 ± 10.16^bx^	40.42 ± 10.95^ABy^
**60 HR**	17.52 ± 10.94^bx^	40.54 ± 10.19^ABy^
**96 HR**	8.71 ± 8.05^cx^	35.88 ± 9.22^By^

Values with different lowercase subscripts (a, b, c) indicate significant differences between timepoints within INRA96 treated samples (*P* < 0.05). Values with different uppercase subscripts (A & B) indicate significant differences between timepoints within OviPlus treated samples (*P* < 0.05). Values with different superscripts (x, y) indicate significant differences between extenders at a given timepoint (*P* < 0.05). Data are presented as mean ± standard deviation (SD).

### Rapid motility

Rapid Motility was significantly higher in INRA96 at timepoint 0 h compared to all other time points (*P* ≤ 0.005) and was significantly decreased at 96 h compared to 12, 24 and 48 h (*P* ≤ 0.01; Table 7 and Fig. 6C). Semen extended in OviPlus exhibited significantly higher rapid motility at timepoint 12 compared to 96 (*P* = 0.0002). Between treatments, rapid motility was significantly higher in OviPlus extended semen compared to INRA96 at all time points beyond 0 h (*P* < 0.0001).

**Table 7:** Rapid motility.

	INRA96 (%)	OviPlus (%)
**0 HR**	31.78 ± 11.28^a^	30.25 ± 11.77^Ab^
**12 HR**	15.36 ± 5.42^bx^	32.94 ± 8.95^Ay^
**24 HR**	15.63 ± 8.20^bx^	29.61 ± 7.38^ABy^
**36 HR**	12.81 ± 7.10^bx^	29.50 ± 8.70^ABy^
**48 HR**	13.21 ± 7.42^bx^	25.22 ± 9.23^ABy^
**60 HR**	10.16 ± 6.91^bcx^	25.42 ± 8.66^ABy^
**96 HR**	5.05 ± 5.16^cx^	21.74 ± 7.80^By^

Values with different lowercase subscripts (a, b, c) indicate significant differences between timepoints within INRA96 treated samples (*P* < 0.05). Values with different uppercase subscripts (A & B) indicate significant differences between timepoints within OviPlus treated samples (*P* < 0.05). Values with different superscripts (x, y) indicate significant differences between extenders at a given timepoint (*P* < 0.05). Data are presented as mean ± standard deviation (SD).

### Progressive circular motility

In INRA96 extended semen samples, significantly greater progressive circular motility was recorded at 0 h compared to 48, 60 and 96 h (*P* ≤ 0.008); 12 h compared to 96 h (*P* = 0.0008); and 24 h compared to 60 and 96 h (*P* ≤ 0.01; Table 8 and Fig. 6d). A tendency for increased progressive circular motility at 24 compared to 48 h was observed (*P* = 0.08). No differences were found between times for semen extended in OviPlus. INRA96 had significantly greater progressive circular motility at 0, 12, 24, and 36 h and trended to have greater progressive circular motility at 48 h (*P* ≤ 0.05) compared to OviPlus.

**Table 8:** Progressive circular motility.

	INRA96 (%)	OviPlus (%)
**0 HR**	1.52 ± 0.66^ax^	0.74 ± 0.75^y^
**12 HR**	0.85 ± 0.41^acx^	0.44 ± 0.33^y^
**24 HR**	1.05 ± 0.59^ax^	0.52 ± 0.26^y^
**36 HR**	0.85 ± 0.49^abx^	0.41 ± 0.20^y^
**48 HR**	0.60 ± 0.47^bc^	0.31 ± 0.13
**60 HR**	0.48 ± 0.51^bc^	0.32 ± 0.10
**96 HR**	0.24 ± 0.42^b^	0.22 ± 0.12

Values with different lowercase subscripts (a, b, c) indicate significant differences between timepoints within INRA96 treated samples (*P* < 0.05). Values with different superscripts (x, y) indicate significant differences between extenders at a given timepoint (*P* < 0.05). Data are presented as mean ± standard deviation (SD).

### Slow motility

In INRA96 samples, significantly more sperm exhibited slow motility at 12, 24, and 36 h compared to 96 h (*P* ≤ 0.03), with nonsignificant trends for more slow motility sperm at 0 and 48 h (*P* ≤ 0.08; Table 9 and Fig. 6e). No differences were found in OviPlus. Comparing treatments, OviPlus had significantly more sperm with slow motility at all time points (*P* ≤ 0.01).

**Table 9:** Slow motility.

	INRA96 (%)	OviPlus (%)
**0 HR**	11.25 ± 4.98^abx^	21.04 ± 10.31^y^
**12 HR**	8.66 ± 4.65^ax^	16.69 ± 3.93^y^
**24 HR**	9.24 ± 5.28^ax^	16.31 ± 3.15^y^
**36 HR**	8.37 ± 4.67^ax^	15.82 ± 3.89^y^
**48 HR**	7.45 ± 3.47^abx^	14.89 ± 2.84^y^
**60 HR**	6.88 ± 4.37^abx^	14.80 ± 2.66^y^
**96 HR**	3.41 ± 2.71^bx^	13.93 ± 2.26^y^

Values with different lowercase subscripts (a & b) indicate significant differences between timepoints within INRA96 treated samples (*P* < 0.05). Values with different superscripts (x, y) indicate significant differences between extenders at a given timepoint (*P* < 0.05). Data are presented as mean ± standard deviation (SD).

### Local motility

Sperm cells moving without forward progression were analyzed through local motility measurements. No differences were observed for INRA96 (Table 10 and Fig. 6f). OviPlus exhibited significantly lower local motility at 0 h compared to 36 and 96 h (*P* ≤ 0.02). OviPlus had significantly more local motility than INRA96 at all time points (*P* < 0.0001).

**Table 10:** Local motility.

	INRA96 (%)	OviPlus (%)
**0 HR**	2.90 ± 1.03^x^	5.15 ± 0.84^Ay^
**12 HR**	3.06 ± 1.08^x^	5.60 ± 1.04^Ay^
**24 HR**	3.25 ± 1.17^x^	6.00 ± 0.94^Ay^
**36 HR**	3.42 ± 1.11^x^	6.30 ± 0.92^By^
**48 HR**	3.29 ± 1.27^x^	5.86 ± 0.57^Ay^
**60 HR**	3.51 ± 1.74^x^	6.25 ± 0.65^Ay^
**96 HR**	2.81 ± 1.36^x^	6.76 ± 0.84^By^

Values with different uppercase subscripts (A & B) indicate significant differences between timepoints within OviPlus treated samples (*P* < 0.05). Values with different superscripts (x, y) indicate significant differences between extenders at a given timepoint (*P* < 0.05). Data are presented as mean ± standard deviation (SD).

## Discussion

The present study builds on the established extender literature by integrating ram-specific liquid semen storage, with single-cell, multiparameter phenotyping to resolve the physiological mechanisms underlying extender-dependent differences in ram sperm survival.

In this study, INRA96, a milk-based extender originally developed for equine semen, was employed for its ability to protect spermatozoa through interactions with milk micelles, whereas OviPlus utilized an egg yolk-based formulation specifically designed for small ruminant sperm. Both extender types mitigate the detrimental effects of Binder of Sperm Proteins (BSPs) on sperm storage; however, they operate through distinctly different protective mechanisms (Salamon and Maxwell 2000; Bergeron et al. 2004; Purdy 2006; Manjunath 2018). BSPs serve important functions in sperm capacitation by mediating the removal of cholesterol and choline phospholipids from the sperm plasma membrane (Thérien et al. 1998; Suarez 2008; Kumar et al. 2016). Despite these necessary roles, prolonged exposure to BSPs during storage can lead to excessive lipid efflux, ultimately compromising the viability of liquid-stored sperm.

Egg yolk-based extenders protect sperm against BSP induced lipid efflux primarily through low-density lipoproteins (LDLs), which exhibit a high affinity for BSPs. The sequestration of BSPs by LDLs prevents excessive lipid removal (Bergeron et al. 2004), thereby stabilizing sperm membranes to allow liquid storage. In bulls, semen extended with egg yolk exhibited 50%–80% fewer BSPs bound to sperm compared to semen extended without LDLs, consequently preserving cholesterol and choline phospholipid levels (Bergeron et al. 2004). Conversely, bull semen stored without LDLs exhibited a 40% reduction in sperm cholesterol and choline phospholipid content within 24 h post-ejaculation. Conversely, milk-based extenders protect sperm via protein-protein interactions rather than lipid-protein mechanisms observed with LDLs (Lusignan et al. 2011). Lusignan et al demonstrated comparable protective effects of whole and skimmed milk, despite skim milk’s negligible lipid content. Caseins, which constitute approximately 2.5% of cow milk content, aggregate into micellar structures and bind BSPs, preventing lipid loss and, consequently, stabilizing the sperm membrane (Lusignan et al. 2011).

Cholesterol plays a critical role in membrane stability by inserting between phospholipid hydrocarbon chains, increasing membrane fluidity and preventing phase transitions (Darin-Bennett and White 1977; Quinn 1989; Travis and Kopf 2002; Mocé et al. 2010; Saez et al. 2011). Ram sperm exhibit relatively low cholesterol-to-phospholipid ratios compared to bulls, humans, rabbits, and monkeys (Holt and North 1984), making cholesterol supplementation particularly important for this species. Accordingly, cholesterol supplementation supports cell viability and limits early onset of the acrosome reaction in multiple species, such as boars, bulls, horses, and rams (Mocé et al. 2010; Moraes et al. 2010; Spizziri et al. 2010; Tomás et al. 2011; Kaewkesa et al. 2016). The inclusion of fresh egg yolk in OviPlus contributes a substantial cholesterol concentration. Thus, semen extended in OviPlus demonstrated greater plasma membrane integrity compared to semen extended in INRA96. These findings align with previous observations of superior membrane integrity in tris-based extenders containing egg yolk compared to milk-based extenders in ram semen (Paulenz et al. 2002; Vodička et al. 2022).

Plasma membrane stability is mechanistically linked to acrosomal integrity, as both structures are vulnerable to lipid peroxidation and osmotic stress (Zhang et al. 2024). The acrosome reaction is an exocytotic process ideally initiated upon sperm binding to the zona pellucida, leading to the release of acrosomal enzymes (Bustani and Baiee 2021). If the acrosome is compromised before the sperm reaches the oocyte due to damage or premature capacitation, the sperm’s fertilization potential is lost (Yousef et al. 2020). Treatment effects were evident, with semen extended in INRA96 yielding a significantly higher percentage of sperm with modified acrosomes at all timepoints 12 h and beyond compared to OviPlus. These results are consistent with previous findings showing fewer intact acrosomes for ram sperm stored in a milk-based extender compared to a tris-based egg yolk extender (Stefanov et al. 2015).

While ROS are necessary in controlled amounts for sperm capacitation, acrosome reaction, and hyperactivation, an imbalance between ROS production and antioxidant defenses leads to toxic lipid peroxide formation, compromising sperm viability and motility (Guthrie and Welch 2012; Gualtieri et al. 2021). Sperm cells are nearly devoid of cytoplasmic antioxidant systems, leaving sperm with minimal protection against ROS (Li 1975; Foote et al. 2002). In vivo, sperm are exposed to primarily anaerobic conditions (Foote et al. 2002) and the antioxidant protective environment of the female reproductive tract (Suarez 2008), the threat of ROS is reduced. However, when storing semen, interventions are necessary to reduce ROS to protect sperm.

Our results showed OviPlus better protected sperm cells from ROS imbalance, especially for the first 48 h, potentially explained by the presence of tryptophan and tyrosine in egg yolk, which serve as free radical scavengers (Nimalaratne et al. 2011). Additionally, our results are consistent with the understanding that osmotic stress can induce oxidative stress (Tapia et al. 2012), as OviPlus showed less plasma membrane damage and lower ROS levels.

While zinc-dependent regulation of sperm capacitation has been described in boar, bull, and human sperm (Kerns et al. 2018), its presence, localization patterns, and extender-dependent redistribution during liquid storage have not previously been demonstrated in ram sperm. Zinc ions play a fundamental role in male fertility, with multifaceted roles in sperm development, membrane stability, antioxidant activity, and fertilization processes (Fallah et al. 2018; Allouche-Fitoussi and Breitbart 2020; Kerns et al. 2020). The removal of Zn^2+^ promotes capacitation by increasing membrane fluidity and initiating intracellular signaling pathways (Travis and Kopf 2002; Tapia et al. 2012). This Zn^2+^ efflux is a prerequisite for subsequent acrosomal remodeling, capacitation, and successful fertilization (Beek et al. 2012; Kerns et al. 2018). Zinc localization patterns, or signatures, have been characterized using image-based flow cytometry, revealing distinct capacitation states (Kerns et al. 2018). This study observed a time-dependent decrease in total sperm Zn^2+^ levels. By 96 h, OviPlus samples retained significantly more Zn^2+^, as evidenced by a larger proportion of Signature 2 cells, in earlier stages of capacitation, with better-preserved fertilization potential.

Sperm motility is an essential fertility predictor in livestock production (Bae et al. 2024). A decline in total, progressive, rapid, and progressive circular motility was observed during 96 h of liquid storage. However, OviPlus exhibited greater stability, with a steady decline in motility, whereas INRA96 showed a significant drop in total, progressive, and rapid motility within the first 12 h. These results are consistent with Stefanov et al who observed a 75% reduction in total motility within 24 h when stored without egg yolk as compared to progressive motility being maintained at 60% of its initial value for 72 h when stored in a Tris-based egg yolk extender (Stefanov et al. 2015). Additionally, Almeida Filho et al recently demonstrated superior progressive motility, coupled with higher plasma and acrosomal membrane integrity in ram semen extended with egg yolk-based diluents (de Almeida Filho et al. 2025).

This study demonstrates that extender composition has profound and coordinated effects on ram sperm physiology during liquid storage. Egg yolk-based OviPlus consistently preserved plasma membrane and acrosomal integrity, delayed oxidative stress, stabilized zinc ion signatures associated with non-capacitated sperm, and maintained motility over extended refrigeration. In contrast, the milk-based extender INRA96 was associated with accelerated membrane destabilization, increased ROS accumulation, premature zinc redistribution, and rapid motility decline, indicative of early capacitation-like changes. By integrating single-cell, multiparameter phenotyping with ram-relevant liquid semen storage, this work provides mechanistic insight into extender-dependent sperm preservation that extends beyond traditional bulk endpoints. These findings support the use of egg yolk-based extenders for fresh ram semen storage and establish a framework for applying high-resolution sperm phenotyping to optimize reproductive technologies in ovine systems.

## List of Abbreviations

AI: artificial insemination
ROS: reactive oxygen species
BSP: binders of sperm proteins
PI: propidium iodide
PNA: peanut agglutinin
CASA: computed assisted semen analysis
IBFC: image-based flow cytometry
